# Trend of malaria prevalence in Wolkite health center: an implication towards the elimination of malaria in Ethiopia by 2030

**DOI:** 10.1186/s12936-020-03182-z

**Published:** 2020-03-16

**Authors:** Absra Solomon, Daniel Kahase, Mihret Alemayehu

**Affiliations:** Department of Medical Laboratory Science, College of Medicine and Health Science, Wolkite University, Wolkite, Ethiopia

**Keywords:** Malaria trend, *P. vivax*, *P. falciparum*, Wolkite, Southern Ethiopia

## Abstract

**Background:**

Malaria is one of the main public health challenges in Ethiopia that hinder the productivity and development of the country. In 2018, Ethiopia is on track to minimize the incidence of malaria by 40% as per its 2020 malaria reduction strategy. Currently, Ethiopia is working to eliminate malaria in 2030 by extending the 2020 strategy. Hence, this study aimed to analyze the trend of malaria prevalence in Wolkite health center, Gurage zone, Southern Central Ethiopia from 2015 to 2018.

**Methods:**

A retrospective study was conducted to determine the prevalence of malaria by reviewing the malaria registration laboratory logbook at Wolkite health center from 2015 to 2018. For all patients, blood films were done for the detection of malaria cases. All the socio-demographic data, year, month and malaria data were collected using a predesigned data collection sheet from January to March, 2019.

**Results:**

From a 121,230 clinically malaria suspected patients, the overall prevalence of microscopically confirmed cases were 8.56% (n = 10,379/121,230). *Plasmodium vivax* was the most predominant species accounted for 69.7% (n = 7237/10,379) followed by *Plasmodium falciparum* 29.3% (n = 3044/10,379). Age group > 15 years old were more affected by malaria accounting 54% (n = 5609/10,379) and malaria cases regarding sex were proportional (51.1% of males and 48.3% of female). Among the catchment areas, a higher number of malaria prevalence was recorded in the Wolkite town 66.2% (n = 0.6538/10,379). Higher malaria cases were shown in the season of Spring 29.8% (n = 3096/10,379) while lower cases 20.4% (n = 2123/10,379) were seen in the Winter season.

**Conclusion:**

The prevalence of malaria in Wolkite health center showed a consistent downward trend from the year of 2015–2018. Importantly, the higher prevalence of *P. vivax* seems overlooked in the study area. Therefore, malaria prevention and control strategy should be reinforced to reduce the prevalence of malaria in the study area.

## Background

Despite a rigorous effort done to prevent malaria, it is still a major public health challenge worldwide causing a significant burden of illness and mortality [1]. Globally, an estimated 219 million malaria cases and 435,000 death occurrence have been reported in 2017. African took a higher share from the global malaria burden and *Plasmodium falciparum* is the most predominant species, accounting for 99.7% [2]. The burden of malaria is more devastating in children and pregnant women [3]. Even though the expanded coverage of malaria prevention and case–control service reduced mortality and morbidity, malaria is a major threat especially to sub Saharan African countries including Ethiopia [4].

Around 68% of the Ethiopian population resides in the area below 2000 m altitude which is considered to be at risk of malaria. Thus, malaria is one of the public health challenges in Ethiopia impede the socio-economic development of the country [4, 5]. The transmission of malaria is highly seasonal throughout the country that depends on altitude and climatic variations [2]. *Plasmodium falciparum* and *Plasmodium vivax* are the most dominant malaria parasites in Ethiopia accounting for 60% and 40% of malaria cases respectively [5]. The major responsible vector for malaria transmission in Ethiopia is *Anopheles arabiensis* however in some other areas *A. pharoensis*, *A. funestus*, and *A. nili* are also responsible for the transmission of malaria [6].

Malaria prevention and control program in Ethiopia is guided by the National Malaria Strategic Plan (NMSP) in line with global Roll Back Malaria (RBM) [4]. The current vector control interventions implemented in Ethiopia include insecticide-treated mosquito nets (ITNs), indoor residual spraying (IRS) and mosquito larval source reduction [7]. As stated in the 2015 malaria indicator survey (MIS), above 70% of households in malaria-endemic areas were protected by ITN or IRS. The national malaria guidelines recommend Artemisinin-based combination therapies (ACTs) for the treatment of uncomplicated malaria caused by *P. falciparum* and for *P. vivax* treatment chloroquine remains efficacious in malaria-endemic areas. Whereas, Primaquine is recommended for radical cure of patients with *P. vivax* in non malarious area, however, to the best of author’s knowledge there was no published data that state the current Primaquine usage in Ethiopia [6, 8].

Ethiopia has recently targeted malaria elimination nationwide in 2030 aligned with the World Health Organization (WHO) Global Technical Strategy (GTS) through intensifying the existing malaria control activities [9]. Presently, Ethiopia is on track to achieve the 2020 milestone by reducing the incidence of malaria by 40% [2]. However, the emergence of insecticide resistance, migrant population, emerging chloroquine resistance for *P. vivax*, difficulty in control and elimination of *P. vivax* are the challenging factors for malaria elimination in Ethiopia and worldwide [5, 10].

According to the Gurage zone heath department, a higher prevalence of malaria was recorded in Wolkite, Abshge and Cheha woredas (district) in 2018 [11]. Analyzing the prevalence of malaria trends each year is important for expanding the prevention and control strategy as well as for designing new policies for appropriate intervention that could assist in the elimination of malaria in 2030. Thus, this study aimed to assess the trend of malaria prevalence in Wolkite health center in Gurage zone from 2015 to 2018. The findings may insight stakeholders to revise malaria control strategies in the study area.

## Methods

### Study area and study population

**Fig. 1:**
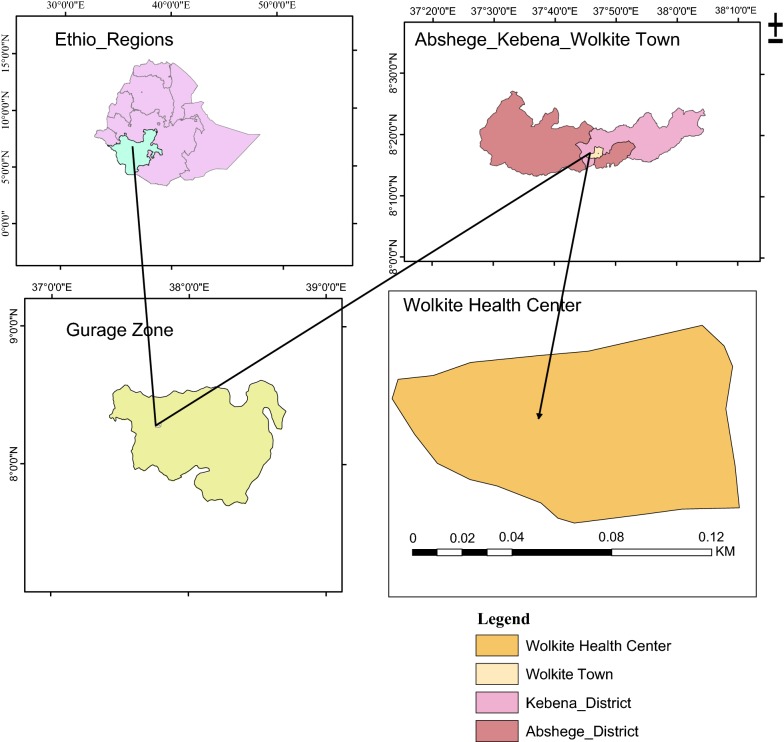
Map of the study area

The study was conducted in Wolkite health center, located in Gurage Zone. Wolkite town is located 158 km southwest of Addis Ababa, the capital city of Ethiopia. Wolkite is the capital city of Gurage Zone that has an average annual temperature of 18.6 °C and an average rainfall of 1244 mm (Fig. 1). The town has an elevation between 1910 and 1935 meters above sea level [12, 13]. Based on the 2007 Census conducted by the Central Statistical Agency of Ethiopia, Wolkite town has a total population of 28,856 of whom 15,068 were males and 13,788 females [14]. Wolkite health center offers diagnosis and treatment for patients that reside in Wolkite town and nearby neighbor woredas like Kebena, Abeshge and others.

Malaria transmission in Gurage Zone is unstable, seasonal and depends on altitude and rainfall. There are two main seasons for transmission of the disease; September to December, after the heavy summer rains and March to May, after the light rains. As the area is malaria-endemic, those individuals who were febrile and had a body temperature above 380 C, were tested for malaria. Thus, all patient results registered for the last 4 years (2015–2018) in the malaria logbook at Wolkite health center were the study population. The data was collected from the health center from January to March 2019.

### Study design

A retrospective study was conducted to determine the trend of malaria prevalence by reviewing the blood film malaria registration laboratory logbook at Wolkite health center Gurage zone, Southern Central Ethiopia from 2015 to 2018.

### Data collection and data analysis

A 4-year malaria data were extracted from laboratory logbook by using data collection sheet including year, month, sex, age, residence, total number of BF examined and species type (*P. falciparum*, *P. vivax* and mixed infections). In Wolkite health center, microscopy is used as a golden standard to confirm the presence of Plasmodium parasite and species identification. From clinically suspected patients, a capillary blood sample was collected to prepare both thin and thick blood films according to standard WHO protocol. After properly labeled and air-dried in a horizontal position, thin films were fixed with absolute methanol and both thin and thick films were stained with 3% Giemsa solution for 30 min. Then blood films were examined microscopically for parasite using 100× oil immersion. If no parasites were found after examining 100 fields, it was reported as negative [15]. In addition, laboratory technologists/technicians involved in malaria microscopy had 2–13 years of experience and well trained for malaria microscopy at least twice in their career.

After checking the data completeness, it was analyzed using SPSS version 20 software. Descriptive statistics were used to analyze microscopically confirmed malaria cases with year. Specifically, the distribution of malaria species in each year with clinically examined and confirmed cases was summarized using a table. Also, Pearson’s Chi-square test was used to determine the association of Plasmodium species with age group, sex and residence. Malaria prevalence was computed by dividing the number of people who showed infection with Plasmodium species to the total number of people examined from the study population. Graphs were used to depict the overall trend of malaria prevalence and malaria species distribution with residence and season. A *P*-value < 0.05 was taken as statistically significant.

## Results

### General characteristics of the Study Population

During the last 4 years, from January 2015 to December 2018, a total of 121,230 patients who were clinically suspected of malaria have been examined for malaria parasites. Of the total patients who requested for blood films, examined blood film, 8.56% (10,379/121,230) were microscopically confirmed for malaria infection which was the overall malaria prevalence. Of these, 5336 (51.1%) were male and 5043 (48.3%) were female. Majority of the study participant was in the > 15 years’ age group and reside in Wolkite town 5609 (54%) and 6555 (62.8%) respectively (Table 1).

**Table 1:** Socio-demographic distribution among the study population in Wolkite health center from 2015 to 2018

Variables (n = 10,379)	Number (%)	Plasmodium species			P value
		*Plasmodium falciparum*	*Plasmodium. vivax*	Mixed infection	
Sex					
Male	5336 (51.1%)	1588 (29.8%)	3700 (%)	48 (%)	0.55
Female	5043 (48.3%)	1456 (28.9%)	3537 (70.1%)	50 (1%)	
Age group (years)					
< 1	516 (5%)	129 (25%)	382 (74%)	5 (1%)	0.02*
1–4	1817 (17.5%)	476 (26.2%)	1321 (72.7%)	20 (1.1%)	
5–14	2437 (23.5%)	717 (29.4%)	1703 (69.9%)	17 (0.7%)	
> 15	5609 (54%)	1722 (30.7%)	3831 (68.3%)	56 (1%)	
Residence					
Abeshge	2934 (28.3%)	1079 (36.8%)	1826 (62.2%)	29 (1%)	< 0.001*
Kebena	607 (5.8%)	175 (28.8%)	428 (70.5%)	4 (0.7%)	
Wolkite	6538 (62.9%)	1687 (25.8%)	4791 (73.3%)	60 (0.9%)	
Other	300 (2.9)	103 (34.3%)	192 (64%)	5 (1.7%)	

* Statistically significant at P < 0.05

### Malaria prevalence at Wolkite health center

**Fig. 2:**
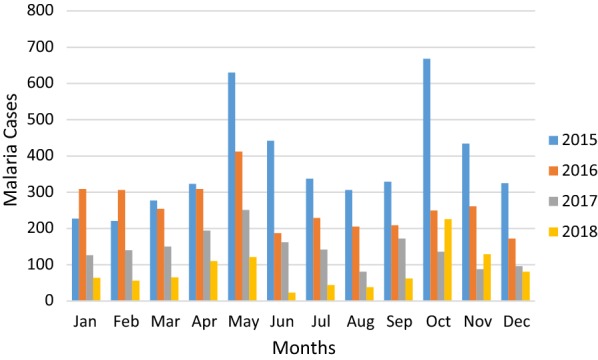
The distribution of confirmed malaria cases by months at Wolkite health center from 2015 to 2018

**Fig. 3:**
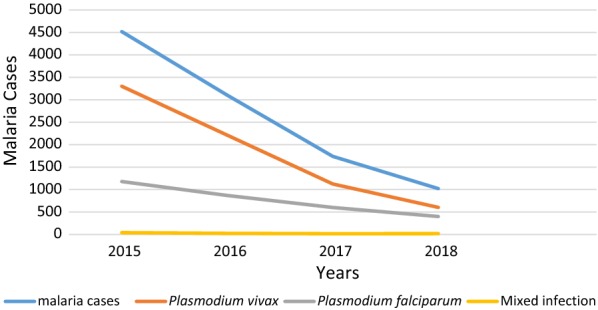
Overall trend of malaria prevalence at Wolkite health center from 2015 to 2018

Of the total 121,230 clinically malaria suspected patients, the utmost number of confirmed annual malaria cases was reported in 2015 and a minimum in 2018 (Fig. 2). Of the Plasmodium species identified, *P. vivax* was the most predominant species accounted for 69.7% followed by *P. falciparum* 29.3% and mixed infection accounted for 0.9% from the confirmed malaria cases (Table 2). In general, consistent reduction of malaria trend was recorded and the slide positivity was 13.5%, 9.8%, 5.5% and 4.1% during the last 4 years from 2015 to 2018 respectively (Fig. 3). All Plasmodium species infections including mixed infection showed a decreasing trend throughout all 4 years. Nevertheless, there was a slight increment in the number of mixed malaria infections in 2018 compared to 2017.

**Table 2:** Malaria case distribution in Wolkite health center from 2015–2018

Year	Microscopically confirmed malaria cases			Clinically examined	Confirmed cases	Slide positivity rate (%)
	*Plasmodium falciparum* positive (%)	*Plasmodium Vivax* positive (%)	Mixed infection (%)			
2015	1179 (26.0%)	3300 (73.0%)	40 (0.9%)	33,543	4519	13.5
2016	867 (27.9%)	2212 (71.2%)	24 (0.8%)	31,527	3103	9.8
2017	599 (34.4%)	1123 (64.6%)	16 (0.9%)	31,441	1738	5.5
2018	399 (39.1%)	602 (59.0%)	18 (1.76%)	24,719	1019	4.1
Total	3044 (29.3%)	7237 (69.7%)	98 (0.9%)	121,230	10,379	8.5

The annual parasitic incidence (API) cases showed a decreasing trend during the last 4 years (2015–2018) 157, 107, 60 and 35 per 1000 population respectively. Similarly, the API for each species has depicted a similar decreasing pattern. The annual falciparum incidence was 41, 30, 21 and 14 per 1000 population whereas annual vivax incidence was 114, 77, 39 and 21 per 1000 population from 2015 to 2018 respectively (Table 3).

**Table 3:** Annual Parasite Incidence in Wolkite health center from 2015–2018

Year	Confirmed malaria case	API	*Plasmodium falciparum* positive	AFI	*Plasmodium vivax* positive	AVI
2015	4519	157	1179	41	3300	114
2016	3103	107	867	30	2212	77
2017	1738	60	599	21	1123	39
2018	1019	35	399	14	602	21
Total	10,379		3044		7237	

*API* annual parasitic incidence, *AFI* annual *falciparum* incidence, *AVI* annual *vivax* incidence

Though malaria cases were detected in all age group the highest affected age group was > 15 years old which accounted for 54% (5609/10,379) followed by 5–14 age group 23.5% (2437/10,379) while the least affected age group was < 1 year 4.97% (516/10,379). In all age groups, *P. vivax* was the most frequently reported species. There was a statistically significant association between Plasmodium species and age groups (P = 0.002). With regard to the sex, males and females were equally infected with a little variation 51.4% and 48.6% respectively. There was no statistical significant association between Plasmodium species and Sex (P = 0.55) (Table 1).

### Malaria species distribution among residence at Wolkite health center

**Fig. 4:**
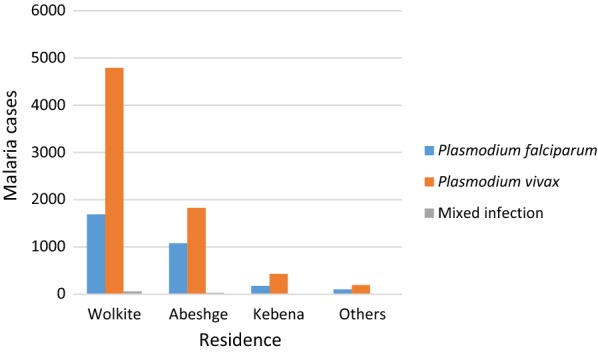
Malaria species distribution among residence at Wolkite health center from 2015 to 2018

*Plasmodium vivax* was the predominant species in all residence and a higher percentage is accounted in Wolkite town 66.2% (6538/10,379) followed by Abeshge Woreda 25.2% (2934/10379), Kebena 5.9% (607/10,379) and 2.7% (300/10,379) in the other. In the meantime, *P. falciparum* was significantly higher in all woredas, accounting 55.4%, 35.4%, 5.7%, 3.4% in Wolkite, Abeshge, Kebena, and other respectively (Fig. 4). There was a statistical significant association between Plasmodium species and residence (P < 0.001).

### Seasonal variation of malaria at Wolkite health center

**Fig. 5:**
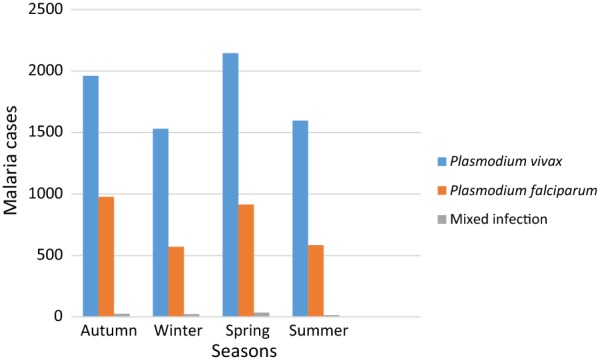
Distribution of Plasmodium species in seasons at Wolkite Health center from 2015 to 2018

The prevalence of malaria for the four seasons in Ethiopia was analyzed. During all the 4 years, May and November were the highest blood film examined accounted 9.9% (11,979/121,230) and 9.5% (11,586/121,230) respectively while the lowest was in July 6.7% (8139/121,230). The maximum number of confirmed malaria cases was reported in spring (September, October, and November) and the minimum was reported during winter (December, January, and February) seasons. *Plasmodium vivax*, *P. falciparum* and mixed malaria infections were higher in spring and lower during the Winter season (Fig. 5) There was no statistical significant association between Plasmodium species and season (P = 0.23).

## Discussion

In the present study, the overall microscopically confirmed malaria cases in Wolkite health center was 10,379 (8.56%) from 2015 to 2018. The maximum and minimum confirmed malaria case was reported in 2015 and 2018 respectively. However, the prevalence was lower than reported from other retrospective studies conducted in Adi Arkay health center (36.1%), Serbo health center (43.8%), Halaba special district (9.5%) and Wolaita zone (33.27%) [16–19]. This variation might be due to climatic and altitude differences. In the previous study, endemic areas of Gurage zone had a good practice of malaria prevention measures accounted for 62% [20]. Moreover, according to 2018 Gurage zone health bureau report, the overall coverage of main malaria interventions, ITNs and IRS, has reached 88% in Gurage zone where Wolkite town accounted 95.6%, Abshge 87%, Kebena 95.5% which attributed for the reduction of malaria incidence [11]. In the study area, *P. vivax* was the dominant species among the Plasmodium species (69.7%) followed by *P. falciparum* (29.3%) during the last 4 years. This was similar to reports from Jimma and Butajira area, near the study area [21, 22]. The biological character of the Plasmodium species including the dormant stage of the parasite in the liver causing relapsing attribute to the predominance of *P. vivax*. In addition, the performance difference of the laboratory personnel regarding malaria species identification might be attributed to increased prevalence. However, despite emerging chloroquine resistance *P. vivax* has been reported in southern Ethiopia [10], chloroquine is an efficacious drug for the treatment of *P. vivax* malaria in the study area [23].

Malaria prevalence trend showed consistent reduction during the last 4 consecutive years from 2015 to 2018 which was in agreement with a study reported in Kenya [24]. All Plasmodium species infections including mixed infection showed a decreasing trend throughout all 4 years. With the exception, a slight increment in the number of mixed malaria infection was recorded in 2018 compared to 2017. This shows continuous efforts were done in scaling up malaria prevention and control at the national and local levels. This might highlight the need for continuous efforts to eliminate malaria earlier than its plan.

In the present study, Plasmodium infection was nearly equal with only slight variation among males and females which was comparable with a study conducted in Wolaita zone [19]. This finding was in contrast with the majority of the study done in Ethiopia [25, 26] where males were highly affected by malaria than females. Both males and females are involved in outdoor activities especially males in the agricultural activities and most females travel to town for the market thus, they are exposed to the bite of Anopheles mosquitoes.

Regarding age, the highest affected age group was > 15 years accounted for 54% followed by 5–14 age group 23.5% while the least affected age group was < 1 year in both sexes and the most frequently reported species in all age groups were *P. vivax*. The highest malaria prevalence in the adult age group obtained was in agreement with other findings reported across Ethiopia [19, 22, 27, 28]. Whereas a finding from Arsi Negelle [29] showed that highly affected age group was 0–5 years followed by 16–20 years old. The reason for this variation might be the majority of the study participants were adults and as this age group is young and productive, they are actively engaged in an agricultural activity that might prone them for an anopheles mosquito bite. In addition, they travel far for schooling which exposes them to the infection.

The prevalence and magnitude of malaria transmission are mainly determined by environmental, climatic and seasonal factors. In highland-fringe areas, like Wolkite town, malaria transmission is seasonal and depends on altitude and rainfall. Thus, higher malaria transmission is recorded after the heavy rain of summer, September to December and the lowest in March to May after the light rains, which agrees with our findings. This was also in agreement with other studies done in the different parts of Ethiopia [26, 28]. Variability of rainfall and temperature in each season affects the availability of breeding habitats for mosquito vectors, the length of mosquito larvae development and the rate of growth of the malaria parasites inside the vector [30].

The distribution of Plasmodium infections across a community varies in a predictable pattern based on age and transmission intensity [31]. A higher prevalence of malaria infection was recorded in Wolkite town followed by Abeshge, Kebena and others. *Plasmodium vivax* was the predominant species in all residences. This variability in the prevalence of malaria among residences agrees with studies undertaken in Butajira [22], Tanzania [32] and Ghana [33]. This might be due to variation in the intervention of malaria prevention and control activities from one area to another. In the present study, there was a statistically significant association of Plasmodium infection with residence and age group. As Plasmodium infection varies between geographical setting and population, the control strategy must vary according to each area’s local epidemiology [31]. Thus, the national malaria control program should enhance the access of malaria prevention to high-risk populations and prioritize high-risk areas based on stratification.

Limitation of the study was poor management of patient data, 46 data were excluded from the study and only 4-year data were available during the data collection time. Since secondary data were used for analysis, it might affect the prevalence of malaria in the study area. However, the present study showed the trend of malaria prevalence in the study area and catchment areas which provides important information to strengthen the intervention of malaria control.

## Conclusion

In general, the prevalence of malaria in Wolkite health center showed a consistent downward trend from the year of 2015–2018. *Plasmodium vivax* seemed to be overlooked even though there were hardly any known drug resistance has been reported in the study area for *P. vivax* [23]. In addition, a recent randomized trial has reported the effectiveness and safety of short-course usage of Primaquine for a radical cure in *P. vivax* endemic areas of Ethiopia [34]. Therefore, malaria prevention and control strategy should be reinforced particularly targeting adults and rural areas and also enhancing the capacity of laboratory personnel in malaria identification is very essential to reduce the prevalence of malaria in the study area.

## Data Availability

The data sets used and/or analyzed during the current study are available from the corresponding author on reasonable request.

## Abbreviations

WHO: World Health Organization
GTS: Global Technical Strategy
SOP: Standard operating procedure
LLINs: Long-lasting insecticide-treated nets
IRS: Indoor residual spraying
SPSS: Statistical Package for Social Sciences
API: Annual parasitic incidence
NMSP: National Malaria Strategic Plan
ACTs: Artemisinin-based combination therapies
RBM: Roll Back Malaria
